# FORMAL CAREGIVER BURDEN IN NURSING ASSISTANTS IN NURSING HOMES: A FEASIBILITY STUDY

**DOI:** 10.1093/geroni/igac059.2055

**Published:** 2022-12-20

**Authors:** Rachel Kunkle, Claudia Chaperon, Ann Berger

**Affiliations:** University of Nebraska Medical Center, Elkhorn, Nebraska, United States; University of Nebraska Medical Center, Omaha, Nebraska, United States; University of Nebraska Medical Center, Omaha, Nebraska, United States

## Abstract

Formal caregivers are the direct care workforce that aid residents in nursing homes. Providing care to residents is hazardous and physically demanding. Formal caregiver burden encompasses five attributes – perceived stress, caring for another, dependency of the older adult, responsibility, and competence. Exploring the five attributes of formal caregiver burden using a mixed-methods approach will determine if the attributes are present and how the nursing home setting contributes to formal caregiver burden. The purpose of this feasibility study is to describe formal caregiver burden of nursing assistants who provide direct care to residents in a nursing home setting in the Midwest United States. Study site one was only able to enroll three participants; therefore, results were limited. However, study site two was able to recruit and enroll the desired sample size (N=9). Interviews and self-report measures (Background/COVID-19 Questionnaire, Perceived Stress Scale, Caring Behaviors Inventory, and Nursing Home Staff Competency Assessment) were completed, and the PI compared integrated mixed methods results. Results suggest all the attributes of formal caregiver burden were present, and no additional attributes were identified. The feasibility of virtual recruitment, enrollment, and data collection procedures were confirmed. Multiple challenges played a role in the unsuccessful recruitment of this feasibility study at site one; however, virtual recruitment was successful at site two. Further exploration will inform identification and measurement of formal caregiver burden to ensure support for nursing assistants, continue the vitality of the nursing assistant workforce in nursing homes/long-term care, and improve the lives of nursing assistants.

